# Patient specific approach to analysis of shear-induced platelet activation in haemodialysis arteriovenous fistula

**DOI:** 10.1371/journal.pone.0272342

**Published:** 2022-10-03

**Authors:** Tatiana Yu Salikhova, Denis M. Pushin, Igor V. Nesterenko, Lyudmila S. Biryukova, Georgy Th Guria

**Affiliations:** 1 National Medical Research Center for Hematology, Moscow, Russia; 2 Moscow Institute of Physics and Technology, Dolgoprudny, Russia; 3 City Clinical Hospital n.a. S.P. Botkin, Moscow, Russia; "INSERM", FRANCE

## Abstract

Shear-induced platelet activation (SIPAct) is an important mechanism of thrombosis initiation under high blood flow. This mechanism relies on the interaction of platelets with the von Willebrand factor (VWF) capable of unfolding under high shear stress. High shear stress occurs in the arteriovenous fistula (AVF) commonly used for haemodialysis. A novel patient-specific approach for the modelling of SIPAct in the AVF was proposed. This enabled us to estimate the SIPAct level via computational fluid dynamics. The suggested approach was applied for the SIPAct analysis in AVF geometries reconstructed from medical images. The approach facilitates the determination of the SIPAct level dependence on both biomechanical (AVF flow rate) and biochemical factors (VWF multimer size). It was found that the dependence of the SIPAct level on the AVF flow rate can be approximated by a power law. The critical flow rate was a decreasing function of the VWF multimer size. Moreover, the critical AVF flow rate highly depended on patient-specific factors, e.g., the vessel geometry. This indicates that the approach may be adopted to elucidate patient-specific thrombosis risk factors in haemodialysis patients.

## Introduction

One of the main functions of platelets is their participation in haemostatic reactions preventing excessive blood loss in case of injury [1,2]. In this respect, a key feature of platelets is their ability to sense high shear flow which occurs at the injury site after primary vasoconstriction [3,4]. This flow-sensing mechanism is based on the interaction of platelets with von Willebrand factor (VWF)–a multimeric protein able to undergo conformational changes under high shear stress [5–7]. Under pathological conditions the same flow-sensing mechanism plays an important role in the initiation of intravascular thrombosis [8–12].

During the recent years substantial efforts have been made to understand shear-induced platelet activation (SIPAct) and its impact on thrombus formation [13,14]. Both experimental (biochemical/biophysical) [15–20] an theoretical (mathematical/computational) [21–25] approaches were found to be very helpful in these efforts. Recent achievements in mathematical modelling [26] combined with current techniques of medical imaging [27,28] and computational fluid dynamics [29,30] open new prospects for patient-specific assessment of the SIPAct. Aim of this research work was to implement such a personalised approach on the example of patients with arteriovenous fistulas (AVF).[6–79]

The AVF is a surgically created connection between an artery and a vein, widely applied in long-term haemodialysis [31,32]. Clinical benefits of its application are primarily limited by the level of thrombotic complications [33,34]. Development of thrombotic risk reduction approaches for patients with AVF is a matter of great concern [35,36]. Increased rate of thrombotic complications among AVF patients might be attributed not only to the surgical procedure itself but also to abnormal high shear flow in AVF [32,37]. In this study, we used clinical imaging data of AVFs obtained from selected patients for computational assessment of the SIPAct level under these abnormal flow conditions.

It is generally accepted that both the magnitude and duration of the shear stress determine whether platelets are activated [16,38,39]. The effect of shear stress on platelets under unsteady flow conditions could be described in terms of its integral characteristic—cumulative shear stress (CSS) [23,40]. If CSS exceeds a certain critical value, SIPAct occurs [16,19]. It has been recently suggested that this threshold value depends on the von Willebrand factor (VWF) multimer size [26].

In the current study, we have combined computational reconstruction of patient-specific vessel geometry and haemodynamics with a recent mathematical model describing VWF-mediated platelet activation [26]. As a result, a novel patient-specific approach for the modelling of SIPAct has been developed. The capabilities of the approach were demonstrated using personalized data of haemodialysis patients with AVFs. It was found that the dependence of the SIPAct level on the AVF flow rate can be approximated by a power law. The critical AVF flow rate was found to be highly depending on patient-specific factors (e.g., the vessel geometry).

We hope that the developed approach may find its further application for the assessment of patient-specific thrombotic risk factors in haemodialysis patients with AVFs. Also the approach might be extended to several other clinically valid settings with increased risk of thrombosis (for example, in circulatory assist devices) [41,42].

## Materials and methods

**Fig 1:**
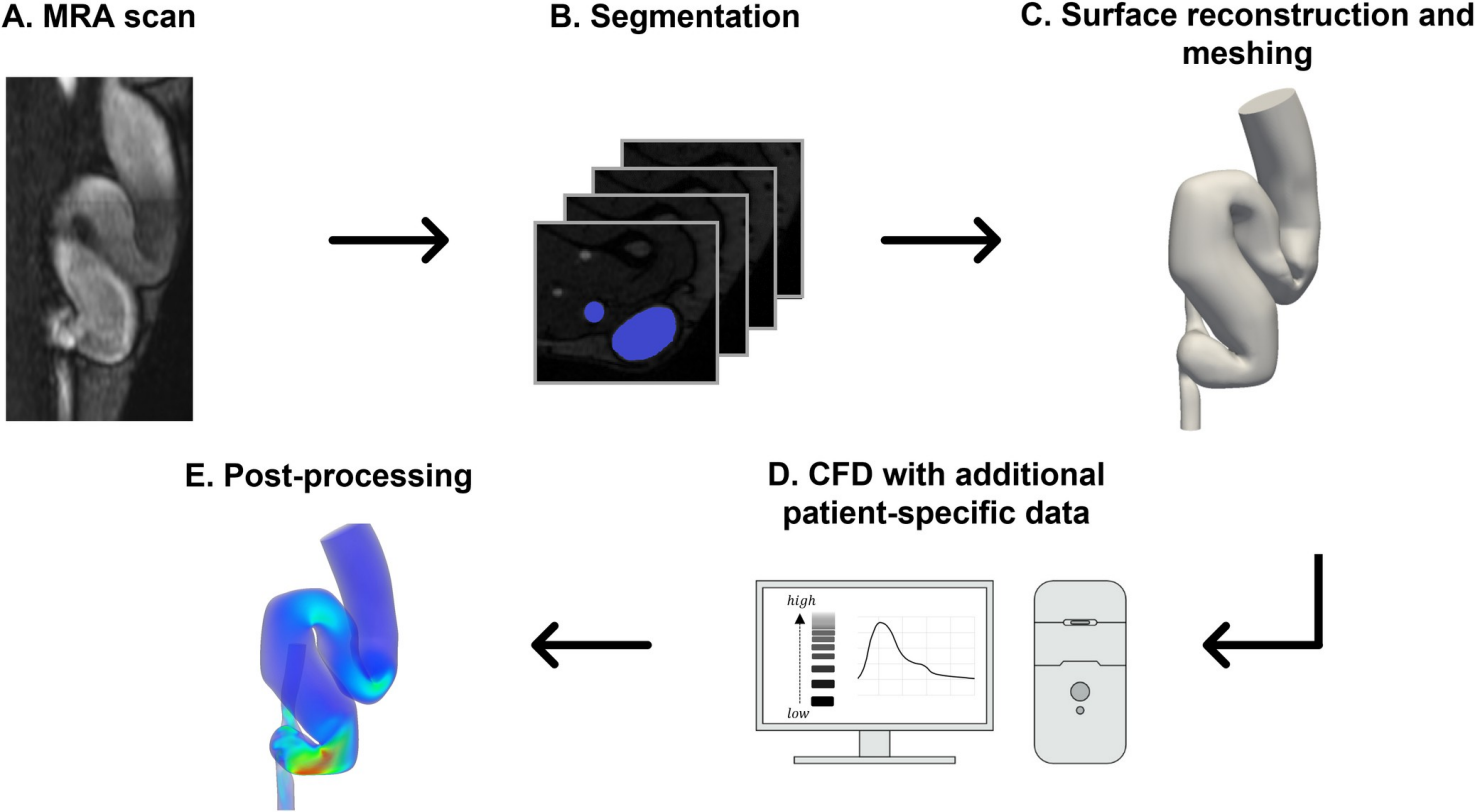
Overview of the SIPAct level estimation in patient-specific AVF geometries. MRA–magnetic resonance angiography; CFD–computational fluid dynamics.

The approach is aimed at evaluating the SIPAct level in the AVF considering patient-specific data. The evaluation is performed via computational fluid dynamics (CFD) methods. Information on the AVF vessel anatomy, blood flow intensity (time-dependent flow waveforms), and VWF multimer size was considered in the calculations (Fig 1). In the current work, patient-specific AVF geometries were used in the calculations. The AVF flow rate and VWF multimer size were chosen as parameters.

### Patient-specific data

Analysis of SIPAct was performed in two mature patient-specific AVFs. The AVF of the first patient (P1) was formed in the forearm of the non-dominant upper extremity from the radial artery and cephalic vein (radiocephalic AVF). The AVF of the second patient (P2) was created at the elbow of the non-dominant arm from the brachial artery and cephalic vein (brachiocephalic AVF). The patients were subjected to non-contrast enhanced magnetic resonance angiography (MRA) of the non-dominant arm vessels. The scan was performed three months postoperatively. MRA images of the arm were acquired with a voxel size of 0.6×0.9×1.4 mm for both AVFs with a 1.5 T scanner (Ingenia, Philips Healthcare, the Netherlands).

The haematocrit was equal to 33% in both patients. Standard coagulogical parameters and additional clinical characteristics of patients are presented at S2 Text.

The study was approved by the Ethics Committee of the National Research Center for Hematology. Serial MRA data of arteriovenous fistulas from 2 patients were acquired at MRI and US diagnostic department of National Research Center for Hematology using protocols approved by the Review Board of National Research Center for Hematology. Written informed consent was obtained from all patients of this study in accordance with the Declaration of Helsinki.

### Patient-specific AVF geometries

**Fig 2:**
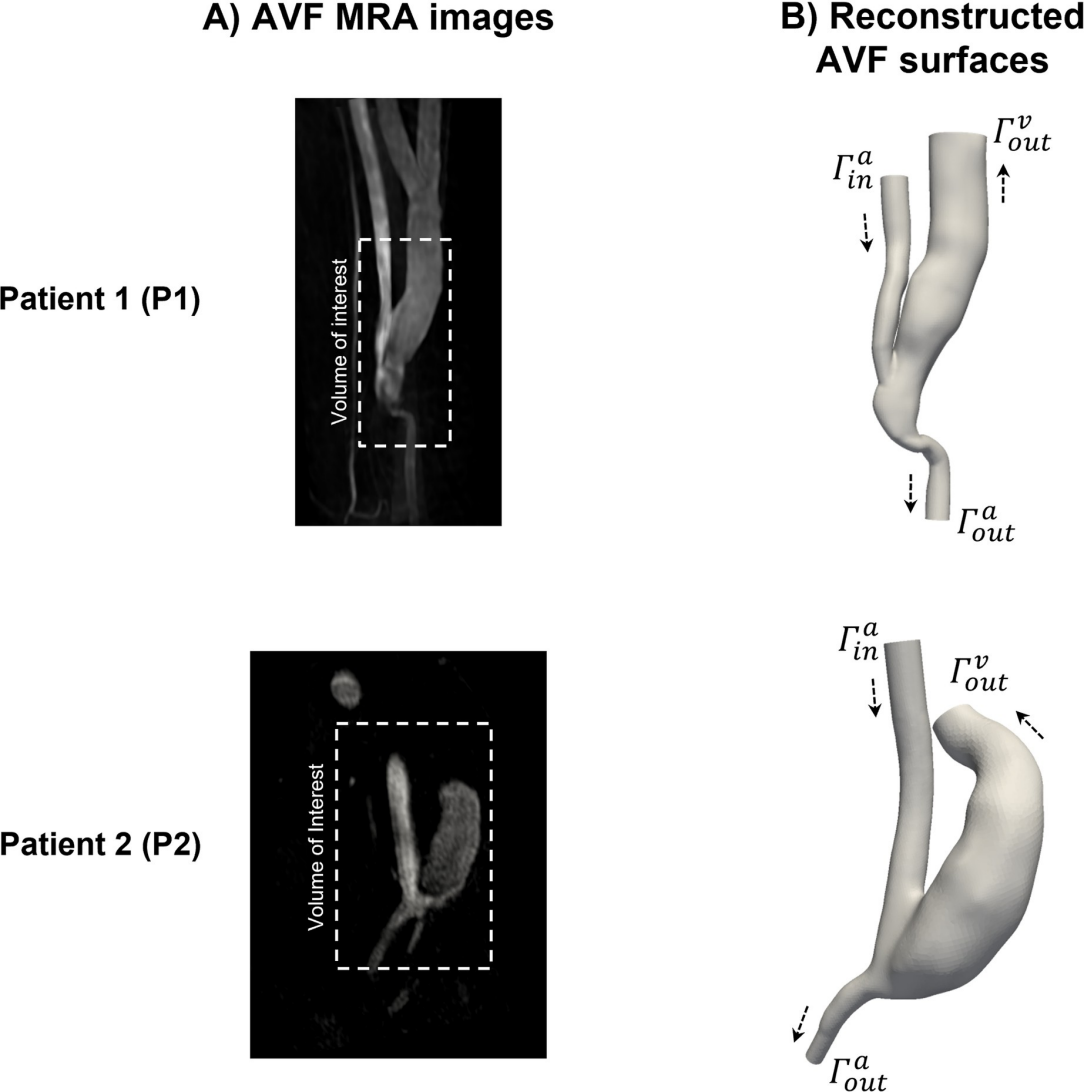
Patient-specific AVF geometries. The left column (A) shows raw non-contrast enhanced MRA data. The right column (B) shows reconstructed three-dimensional AVF geometries. The white dashed rectangle in the raw MRA data delineates the zone of interest. The black arrows indicate the direction of blood flow in the AVF vessels. Blood flows into the AVF through the artery inlet ($\Gamma_{in}^{a}$) and leaves the AVF through the artery ($\Gamma_{out}^{a}$) and vein ($\Gamma_{out}^{v}$) outlets in all CFD calculations. The P1 and P2 symbols designate the AVFs of the first and second patients, respectively.

Patient-specific geometries of the AVFs were reconstructed from MRA data via methods described elsewhere (Fig 2) [43]. Straight cylindrical extensions with a length of at least one diameter of the adjacent vessel were added to AVF vessels to ensure flow development [44]. A hexahedral volume mesh was generated based on the reconstructed geometries. Four layers of thin prism cells with a growth rate of 1.2 inwards were placed near the AVF vessel walls to resolve the boundary layer [45]. The grid convergence index method was adopted to determine the appropriate cell number in the computational meshes (S1 Text) [46]. The final volume meshes contained approximately 930000 (AVF P1) and 950000 (AVF P2) cells with characteristic sizes of 0.016 mm and 0.024 mm, respectively.

### Basic equations

This work is aimed at the analysis of platelet activation initiated by the unfolding of VWF multimers on platelet surfaces [24,26]. VWF unfolding is supposedly initiated under overcritical cumulative shear stress [26]. The subsequent binding of VWF multimers with platelet GPIb receptors triggers outside-in signalling, i.e., platelet priming [47,48]. Platelet priming leads to platelet activation at which conspicuous functional consequences (e.g., P-selection expression) are observed [19]. In this regard, the SIPAct level in the investigated region can be estimated via the calculation of the priming platelet number [49].

The calculation of the SIPAct level was performed in several steps. First, unsteady Navier-Stokes equations were solved [50]. As a result, the distributions of the velocity and static pressure in the AVFs were obtained. Blood was treated as an incompressible Newtonian fluid. The dynamic viscosity was calculated via its well-established dependence on the haematocrit [51]. A time-dependent Poiseuille velocity profile was prescribed at the inlet ($\Gamma_{in}^{a}$, Fig 2) and outlet ($\Gamma_{out}^{a}$, Fig 2) of the artery. The instantaneous average velocity was calculated based on the volumetric flow rate. Volumetric flow rate waveforms were adapted from the literature [52]. The static pressure remained fixed, and velocity was allowed to float at the vein outlet ($\Gamma_{out}^{v}$, Fig 2). These boundary conditions are typically considered at the outflow boundary in internal flow calculations [53]. A no-slip condition was prescribed at the vessel walls. The influence of vascular distensibility on the distribution of the variables of interest was assumed to be non-significant (rigid wall assumption) [54,55].

After solving the Navier-Stokes equations, the distribution of the *CSS* in the AVFs was obtained. For this purpose, components of the viscous shear stress tensor in the AVF were determined via the calculated velocity distribution and patient dynamic viscosity [56]. The magnitude of shear stress (*τ*) on platelets at a given point of space was calculated as previously described [56]. The calculated shear stress distribution *τ* was considered in the estimation of the *CSS* in the AVF according to the following equation:

$$\frac{\partial CSS}{\partial t}+(\vec{V},\vec{\nabla })CSS=\tau \cdot \theta (\tau -\tau_{\#})$$ (1)

where $\vec{V}$ denotes the velocity vector, $\vec{\nabla }$ is the nabla operator, *θ*(*τ*−*τ*_#_) denotes the Heaviside function, and *τ*_#_ ≡ *τ*_#_(*N*) denotes the threshold value of the shear stress for the VWF multimers with a given number of monomers (*N*). Eq (1) states that only overcritical shear stress is considered in the cumulative shear stress calculation. The dependence of the shear stress threshold on the VWF multimer size was derived earlier [26]. This dependence is approximated by the following relation at *N* ≫ 1:

$$\tau_{\#}=\tau_{0}\cdot N^{-2/3}$$ (2)

where *τ*_0_ is a dimensional factor. The following set of boundary conditions for Eq (1) was considered. The cumulative shear stress was equal to zero at the artery inlet ($\Gamma_{in}^{a}$, Fig 2). A zero-gradient condition was prescribed at the artery ($\Gamma_{out}^{a}$, Fig 2) and vein ($\Gamma_{out}^{v}$, Fig 2) outlets. This condition is typically applied in modelling of passive scalar transport at the outflow boundary [57]. A no-flux condition was prescribed at the vessel walls.

The obtained distribution of the *CSS* in the AVF was used to calculate the platelet priming level. First, the following equation was solved:

$$\frac{\partial P}{\partial t}+(\vec{V},\vec{\nabla })P=-kP∙\theta (CSS-CSS_{0})$$ (3)

where *P* denotes the concentration of resting platelets with globule-like VWF multimers grafted onto the platelet surfaces, *CSS*_0_ ≡ *CSS*_0_(*N*) denotes the threshold cumulative shear stress for unfolding of VWF multimers with a given size and *k* is a rate constant. The distribution of the priming platelet concentration (*P*_*a*_) was further determined according to the following balance condition:

$$P_{a}=P_{0}-P$$ (4)

where *P*_0_ is the initial resting platelet concentration. The value of *CSS*_0_ for a given *N* in Eq (3) was calculated via the explicit expression derived previously (S2 Text) [26]. The dependence may be approximated at *N* ≫ 1 with the expression:

$$CSS_{0}\approx C∙N^{1/3}$$ (5)

where *C* is a dimensional factor. *P* was set to *P*_0_ at the artery inlet ($\Gamma_{in}^{a}$, Fig 2). A zero-gradient condition was prescribed at the artery ($\Gamma_{out}^{a}$, Fig 2) and vein ($\Gamma_{out}^{v}$, Fig 2) outlets. A no-flux condition was set at the vessel walls.

The variable of the platelet activation level (PAL) was considered a characteristic of the SIPAct level in the fistulas:

$$PAL=\left(\frac{1}{\Delta t}\int_{t_{0}}^{t_{0}+\Delta t}\frac{J_{a}}{J_{\Sigma }}dt\right)∙100\%$$ (6)

where Δ*t* = *m*Δ*t*_0_, *m* denotes the number of cardiac cycles, Δ*t*_0_ denotes the duration of a cardiac cycle, *J*_*a*_ is the convective flux of the priming platelets, and *J*_Σ_ is the total convective platelet flux expressed as:

$$J_{a}=\oint_{\Gamma_{out}^{v}}P_{a}\vec{V}\cdot \vec{dS}$$ (7)

$$J_{\Sigma }=\oint_{\Gamma_{out}^{v}}P_{0}\vec{V}\cdot \vec{dS}$$ (8)

where $\vec{dS}$ denotes the surface vector of an infinitesimal part of the vein outlet, and *P*_0_ was introduced previously (Eq (4)).

The values of the parameters are given in S2 Text.

### Methodology of SIPAct analysis in the AVF

The aim of the study was to calculate the dependence of the SIPAct level (Eq (6)) on the AVF flow rate and VWF multimer size in patient-specific AVFs (Fig 2).

The influence of the first factor was investigated by varying the average volumetric flow rate $Q_{in}^{a}$ per cardiac cycle at the arterial inlet ($\Gamma_{in}^{a}$, Fig 2). According to clinical studies, the value of $Q_{in}^{a}$ may range from 300 mL/min to 1000 mL/min in successfully mature radiocephalic AVFs [58,59]. In turn, the average flow rate in successfully mature brachiocephalic AVFs typically ranges from 500 mL/min to 1500 mL/min [58,59]. In this work, the upper value of $Q_{in}^{a}$ was set to 775 mL/min and 1350 mL/min for AVF P1 and AVF P2, respectively. The flow rate $Q_{out}^{a}$ through the arterial outlet ($\Gamma_{out}^{a}$, Fig 2) was set to 50 mL/min, and the flow direction was chosen to be anterograde, i.e., directed towards the hand. The lower value of $Q_{in}^{a}$ was set to 150 mL/min as the value of $Q_{out}^{a}$ rarely exceeds one-third of $Q_{in}^{a}$ [60]. The average flow rate through the fistula vein (AVF flow rate) $Q_{v}^{out}$ was considered during post-processing of the calculation results:

$$Q_{out}^{v}=Q_{in}^{a}-Q_{out}^{a}$$ (9)

The time-dependent volumetric flow rate waveforms are given in S3 Text.

The number of monomeric subunits (*N*) in the VWF multimers ranges from 2 to 80 under physiological conditions [61]. VWF multimers with a size smaller than 10 do not practically affect platelets [62–64]. In turn, VWF multimers with more than 80 monomers are detected in blood only under certain pathological conditions [61]. In this work, the value of *N* ranges from 10 to 100 monomers, unless otherwise specified. The VWF size distribution in blood plasma is assumed to be monodisperse.

Preliminary calculations indicated that different numbers of cardiac cycles are needed for the establishment of quasiperiodic oscillations of *J*_*a*_ and *J*_Σ_ (Eqs (7) and (8)) at low and high AVF flow rates, respectively. The duration of the calculations ranged from 6 to 15 s (S4 Text). The limitations of the current approach are discussed in S5 Text.

### Numerical methods and programs

Reconstruction of the patient-specific AVF geometries was performed in SimVascular software [43]. Computational meshes were generated in CF-MESH+ software (№128-14790459). CFD calculations were performed in OpenFOAM software [65]. The Navier-Stokes equations, Eqs (1) and (3), were solved numerically via the finite volume method with splitting techniques [66–68]. To discretize the convective term in the Navier-Stokes equations, a high-resolution scheme was adopted [69]. The upwind scheme was applied to discretize the convective terms in Eqs (1) and (3) [66,70]. The time term in all partial differential equations was discretized via the Crank-Nicholson scheme [71]. An adjusted time step was used, whose size was calculated from the condition of *Co* < 1, where *Co* is the Courant number [30]. The Navier-Stokes equations were solved with the PISO algorithm [72]. A DIC-preconditioned conjugate-gradient (PCG) linear solver was used to calculate the pressure field, and a DILU-preconditioned biconjugate gradient stabilized (BiCGSTAB) linear solver was used for the remaining fields [73]. Visualization of the calculation results was performed in ParaView software [74].

## Results

**Fig 3:**
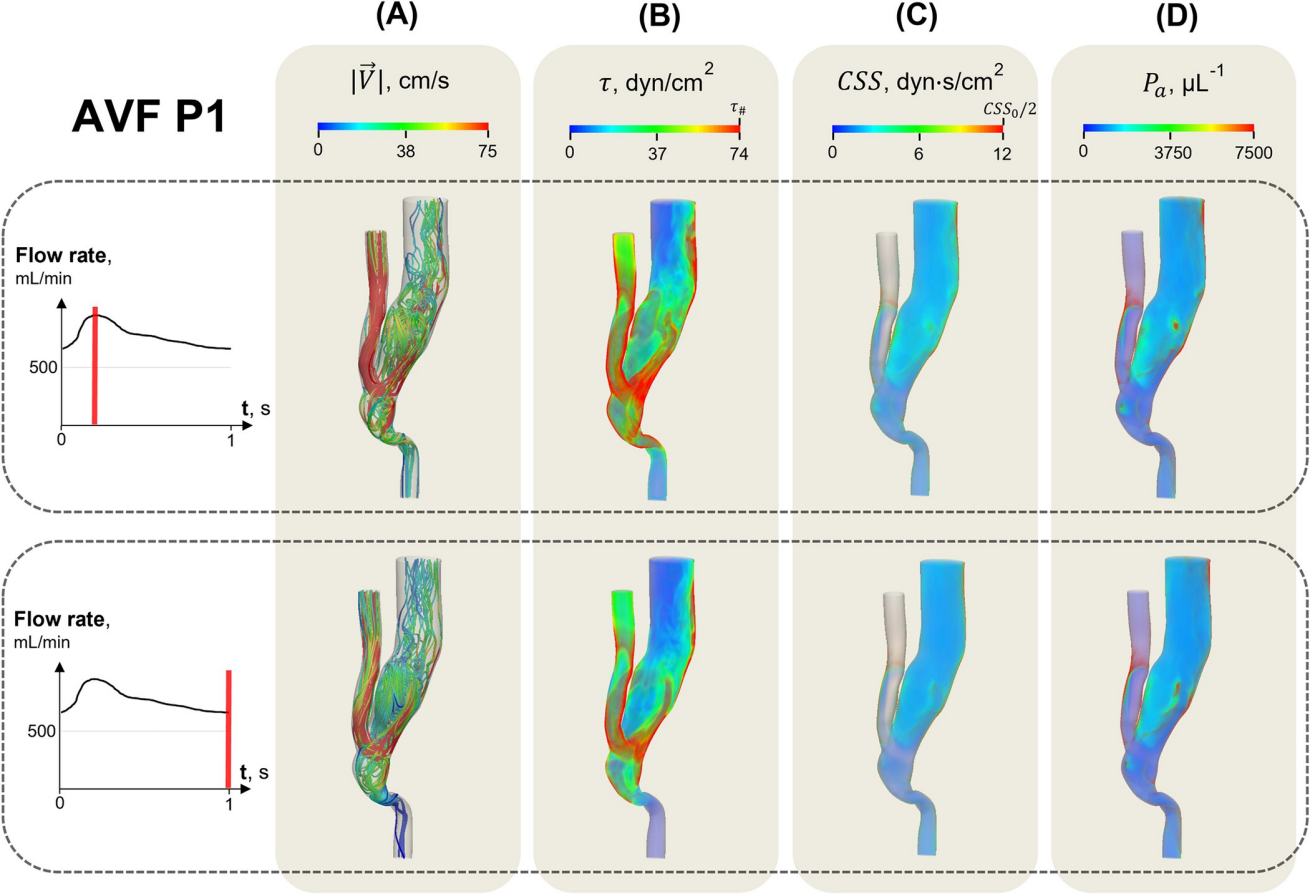
Key calculated variables in the AVF P1 in systole (upper row) and diastole (lower row). The distributions of the velocity magnitude |$\vec{V}$| (A), shear stress *τ* (B), cumulative shear stress *CSS* (C) and primed platelets *P*_*a*_ (D) are obtained at the highest parameter values ($Q_{out}^{v}$ = 725 mL/min, *N* = 100). The blue and red colours correspond to the lowest and highest variable values, respectively. The links for the supporting movies are available in S6 Text.

The distribution of the key variables in the AVF P1 at the different stages of the cardiac cycle is shown in Fig 3 ($Q_{out}^{v}$ = 725 mL/min, *N* = 100). The analysis of the calculated streamline behaviour demonstrated that blood flow in the fistula vein exhibited a complex nature throughout the cardiac cycle (Fig 3A). The flow originating from the proximal part of the artery (i.e., located closer to the heart from anastomosis) formed a recirculation zone along an inner venous wall. The blood flow in this zone was characterized by an irregular change of the direction of the velocity vector. The observed flow unsteadiness additionally indicated that both the amplitude and duration of the shear stress should be considered in the estimation of the blood flow effect on platelets. Zones of overcritical shear stress and cumulative shear stress satisfying the conditions of *τ* > *τ*_#_ and *CSS* > *CSS*_0_, respectively, occurred adjacent to the proximal part of the artery wall and the outer fistula vein wall (Fig 3B and 3C, respectively). The presence of these zones caused platelet priming in the fistula vein throughout the entire cardiac cycle (Fig 3D). The SIPAct level (Eq (6)) did not exceed 2%.

**Fig 4:**
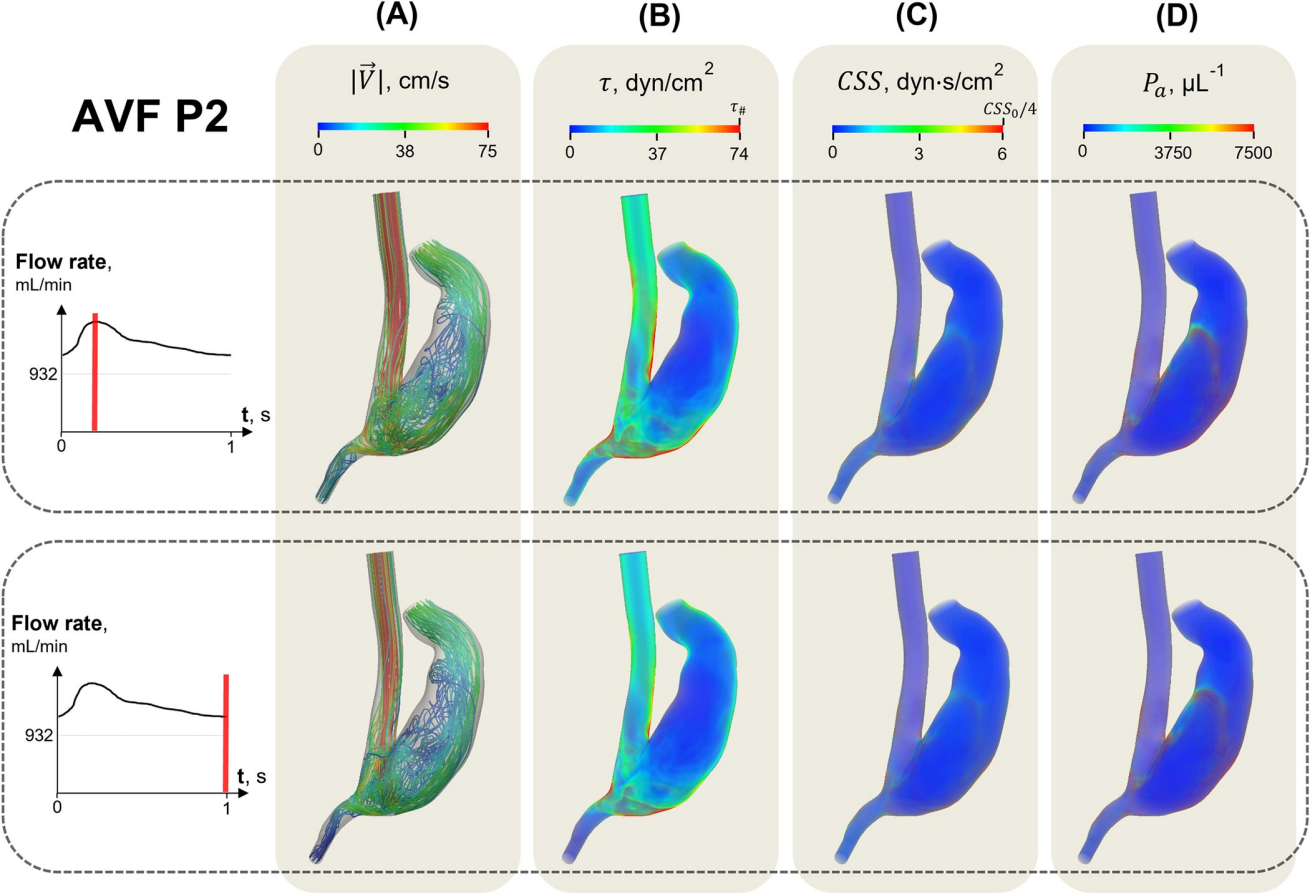
Key calculated variables in the AVF P2 in systole (upper row) and diastole (lower row). The distributions of the velocity magnitude |$\vec{V}$| (A), shear stress *τ* (B), cumulative shear stress *CSS* (C) and primed platelets *P*_*a*_ (D) are obtained at the highest parameter values ($Q_{out}^{v}$ = 1300 mL/min, *N* = 100). The blue and red colours correspond to the lowest and highest variable values, respectively. The links for the supporting movies are available in S6 Text.

The calculation results of SIPAct in AVF P2 (Fig 2) are shown in Fig 4 ($Q_{out}^{v}$ = 1300 mL/min, *N* = 100). The streamline behaviour throughout the cardiac cycle was qualitatively similar to that in AVF P1 (Fig 4A). Zones of overcritical shear stress and cumulative shear stress were observed throughout the entire cardiac cycle (Fig 4B and 4C). The SIPAct level in the AVF P2 did not exceed 0.2%.

**Fig 5:**
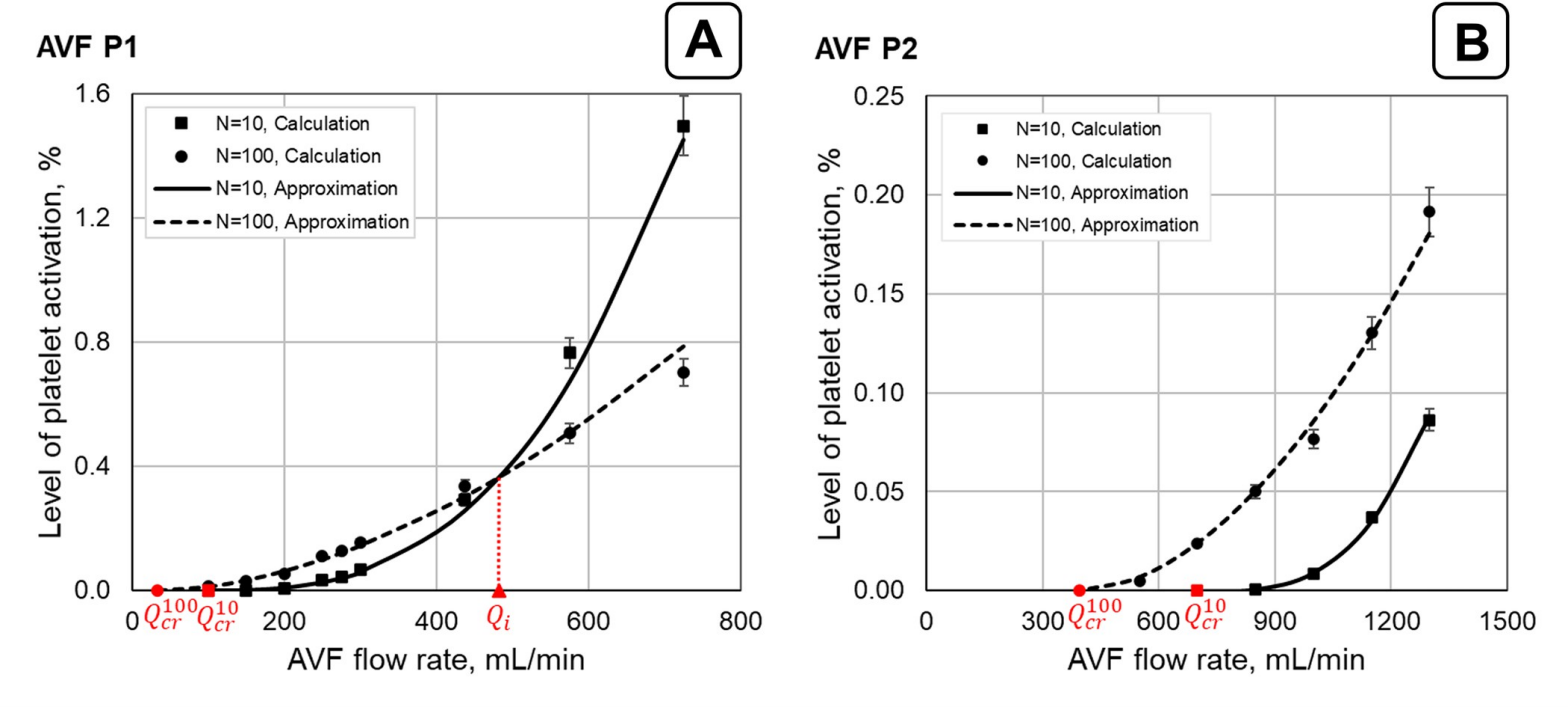
Dependence of the SIPAct level on the flow rate for two multimer sizes in patient-specific AVFs. The calculation results for P1 and P2 AVFs are shown in graphs (A) and (B), respectively. The calculated points are obtained for VWF multimer sizes (*N*) of 10 and 100. The approximation curves (Eq (10)) are represented by the solid (*N* = 10) and dashed (*N* = 100) lines. *Q*_*i*_ denotes the AVF flow rate at which the approximating curves intersect. $Q_{cr}^{10}$ and $Q_{cr}^{100}$ denote the critical values of the flow rate through the fistula vein below which SIPAct was not observed for VWF multimer sizes of 10 and 100, respectively. The calculation results for intermediate values of the VWF multimer size are given in S6 Text.

The influence of the AVF flow rate and VWF multimer size on the level of SIPAct was investigated in both AVFs (Fig 5). The SIPAct level monotonically increased with an increasing flow rate in both AVFs (Fig 5; S6 Text). In the fistula of the first patient, the SIPAct level was equal at a certain value of the flow rate *Q*_*i*_ for the VWF multimer sizes *N* = 10 and *N* = 100 (Fig 5A). The SIPAct level was higher for larger multimer sizes (*N* = 100) at flow rates *Q*_*v*_ < *Q*_*i*_. In the case of *Q*_*v*_ > *Q*_*i*_, the SIPAct level was higher for smaller VWF multimer sizes (*N* = 10).

Notably, the intersection point of *Q*_*i*_ was not observed in the AVF P2 (Fig 5B). The larger the VWF multimer size was, the higher the SIPAct level. Consequently, larger VWF multimers could pose a higher risk of SIPAct over the investigated range of flow rates in AVF P2.

The results (Fig 5) were approximated by an equation with the following form:

$$PAL=a{\left(Q_{v}^{out}-Q_{cr}\right)}^{\beta }$$ (10)

where *a*, *Q*_*cr*_ and *β* are approximation parameters. The exponent *β* was found to be a decreasing function of the VWF multimer size for both patients (Table S6.1 in S6 Text). The obtained result suggests that the SIPAct level for smaller VWF size should exceed the SIPAct level for larger VWF size with an increasing flow rate in AVF P2. In this regard, the point of *Q*_*i*_ seems to lie outside the investigated range of the flow rate (Fig 5B). The abscissa of the curve intersection point (*Q*_*i*_) was equal to approximately 1585 mL/min for AVF P2. This value exceeds the maximum flow value applied in the simulations (1300 mL/min).

The calculations also indicated that the SIPAct level in AVF P1 is nonzero up to the minimum AVF flow rate ($Q_{out}^{v}$ = 100 mL/min, Fig 5A). In this regard, estimation of the critical flow (*Q*_*cr*_, Eq (10)) was performed via the correlation coefficient maximization method [75]. In turn, the critical flow rates in the case of AVF P2 were obtained via the bisection method. The smaller the multimer size was, the higher the critical flow rate for both AVFs. The founded critical flow rates for the AVFs are presented in S6 Text.

**Fig 6:**
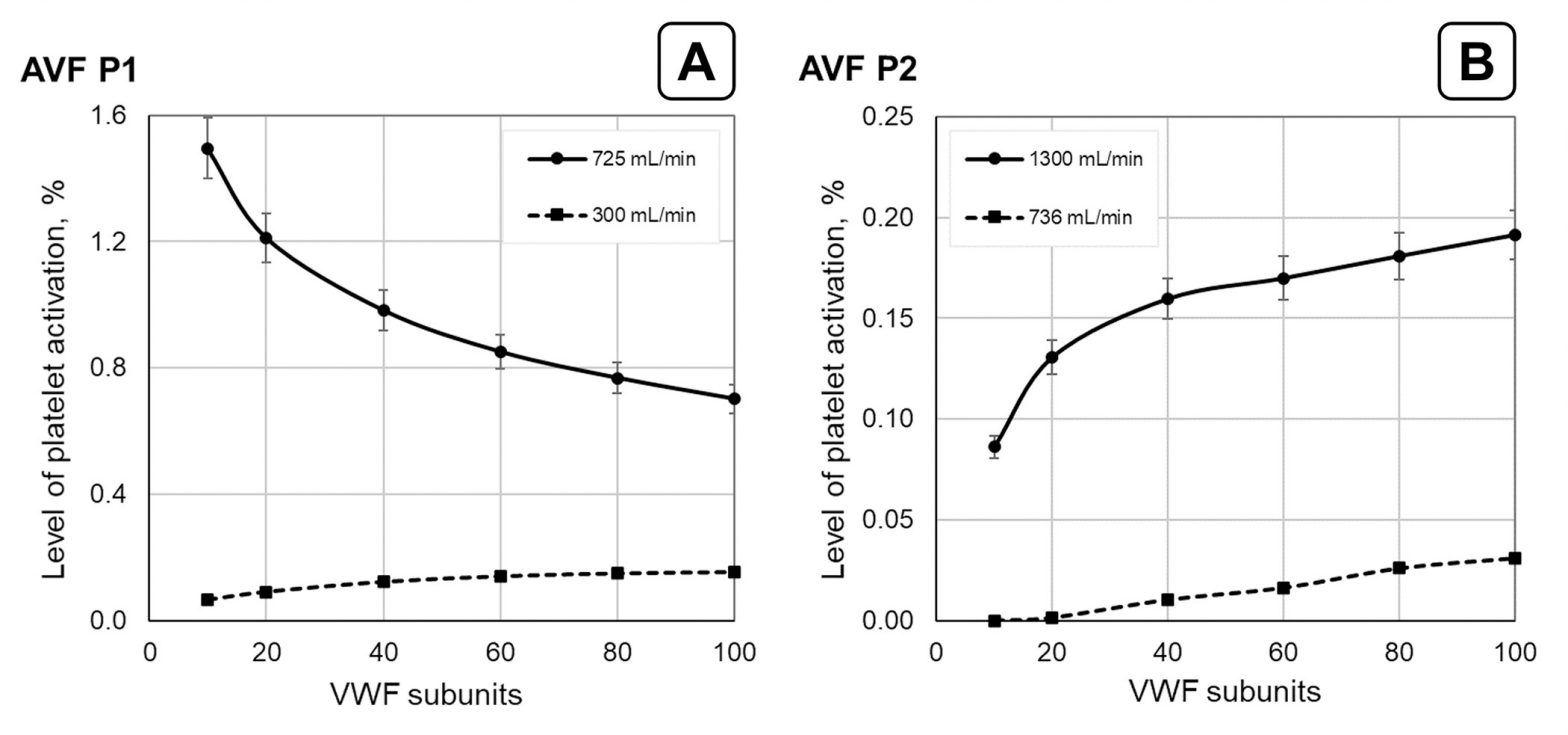
Dependence of the SIPAct level on the VWF multimer size in P1 (A) and P2 (B) AVFs. The results are obtained at AVF flow rates ($Q_{v}^{out},$ Eq (7)) of 300 mL/min (dashed line) and 725 mL/min (solid line) for the first patient. The first value corresponds to the minimum flow rate required for effective haemodialysis. The second value represents the maximum AVF P1 flow rate used in the calculations. The results are obtained at AVF flow rates of 736 mL/min (dashed line) and 1300 mL/min (solid line) for the second patient. The first value corresponds to the flow rate when SIPAct is absent for the lowest VWF multimer size within the considered range (*N* = 10). The second value is the maximum AVF flow rate used in this work for the second patient. The solid and dashed lines are to guide the eye.

The influence of the VWF multimer size on the SIPAct level is shown in Fig 6. The SIPAct level monotonically increased with increasing multimer size at a relatively low flow rate (300 mL/min, Fig 6A). However, the SIPAct level was a decreasing function of the VWF multimer size at a higher flow rate (725 mL/min). This suggests that a reduction in the VWF size may paradoxically lead to an increase in the SIPAct level at sufficiently high flow rates through AVF P1.

**Fig 7:**
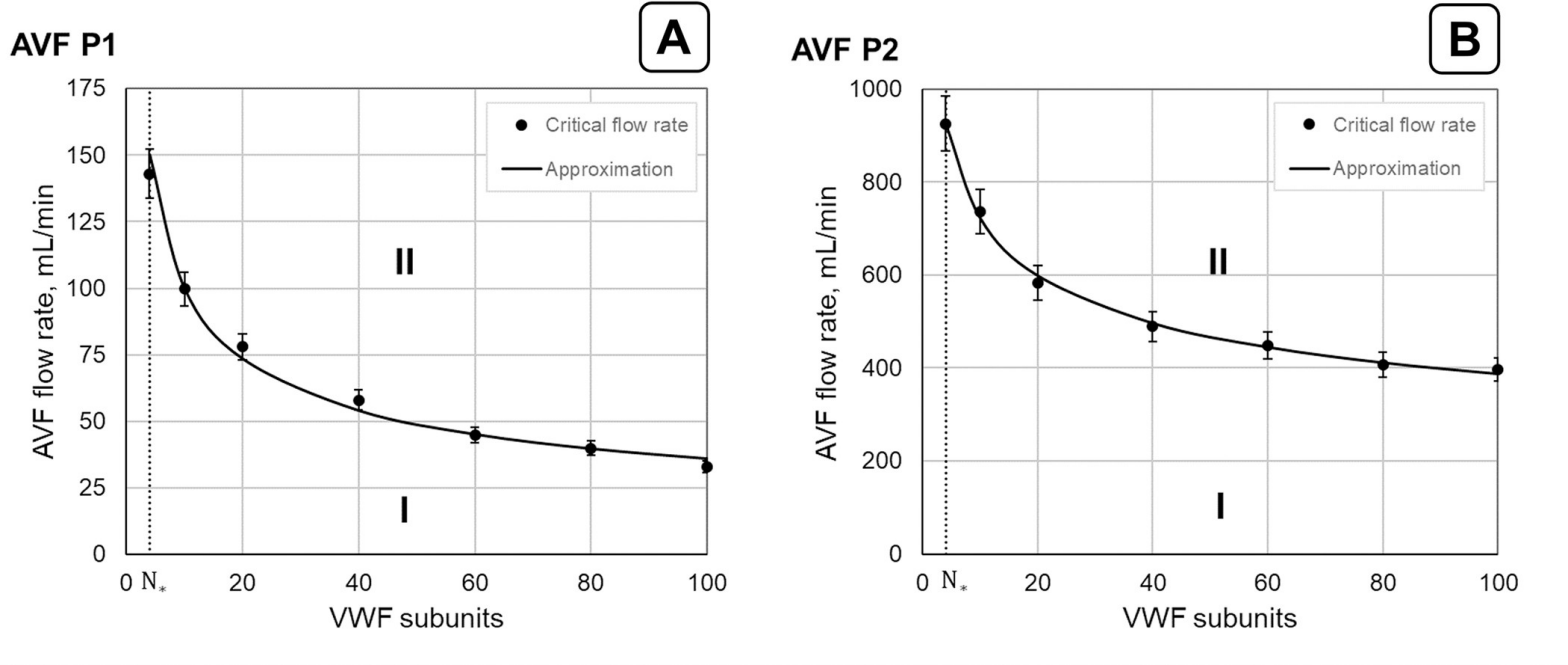
Parametric diagram of SIPAct in P1 (A) and P2 (B) AVFs. Region I corresponds to the values of the parameters at which no SIPAct should be observed. Region II represents the values of the parameters at which SIPAct can occur. The solid lines approximate the dependence of the critical flow rate (*Q*_*cr*_) on the VWF multimer size. The value N_*_ (N_*_ = 4) is the minimum number of VWF monomers when the approach used in this work is applicable [24]. The parameter values of the approximation curves are given in S6 Text.

A parametric diagram of SIPAct in the AVF was obtained for both patients (Fig 7). The dependence of the critical flow rate *Q*_*cr*_ on the VWF multimer size enabled us to distinguish two parameter regions. The first region corresponds to the values of the parameters where no SIPAct was observed (region I). In turn, the second region is the region of the values of the parameters for which SIPAct should occur (region II). The critical flow rate monotonically decreased with increasing VWF multimer size. Thus, a lower AVF flow rate is needed to induce SIPAct at larger VWF multimer sizes in both AVFs.

The AVF should maintain a minimum blood flow of 300 mL/min to sustain effective haemodialysis [76]. The critical flow rates for AVF P1 were significantly lower than the abovementioned value (Fig 7A). This suggests that SIPAct should occur within a practically important flow rate range in this case. In contrast, the SIPAct level remained zero up to flow rates of approximately 740 mL/min ($Q_{cr}^{10}$) and 400 mL/min ($Q_{cr}^{100}$) in the AVF P2 (Fig 7B). It can be concluded that SIPAct is not a thrombosis risk factor for the second patient up to the determined critical flow rates.

## Discussion

In the current work, a patient-specific approach for the modelling of SIPAct in AVFs was developed. This approach is based on the idea that the critical shear stress and critical cumulative shear stress depend on the VWF multimer size (Eqs (2) and (5)) [24,26]. The SIPAct level in the AVF is calculated via CFD methods considering the abovementioned dependencies and realistic geometries of the fistula vessels reconstructed from medical images. The capabilities of the approach for the analysis of SIPAct in patient-specific AVF geometries were demonstrated (Figs 5–7).

Methods for SIPAct modelling via medical imaging and CFD methods began to develop since the early 2000s [77-79]. These approaches were focused on the investigation of biomechanical factors influencing the initiation of thrombus formation. In particular, the presence of overcritical shear stress zones was explored.

In the current work, the SIPAct level dependence on both biomechanical factors (Fig 5) and the VWF multimer size (Fig 6) were analysed. The influence of the VWF multimer size on the SIPAct level in AVF P1 yielded opposite effects at low and high AVF flow rates (Fig 6A). In particular, the SIPAct level increased with decreasing VWF size at high flow rates. This effect was not observed in the AVF P2 (Fig 6B). It was shown that SIPAct in AVF P2 may be completely absent within the physiological range of the VWF multimer size at flow rates sufficient for haemodialysis (Fig 7A). In contrast, SIPAct in AVF P1 should be initiated within the practically important range of the flow rate (Fig 7B).

The obtained results vary for different patients. Thus, patient-specific factors such as the anatomical structure of the AVF vessel and emerging flow abnormalities might be considered as a source of additional risk of thrombotic complications in haemodialysis patients. Moreover, the approach developed in the current paper allows to quantitatively characterize these individual factors from the point of view of potential platelet activation risk. Further studies on sufficiently large cohorts of patients will be necessary to determine clinical prognostic value of the developed approach [80,81]. Also, a careful correlation analysis including the results of numerical simulations and different other biomarkers (e.g. sP-selectin, GPIIb-IIIa) will be needed to determine the relative impact of SIPAct on the thrombosis risk in haemodialysis patients. Such a broad-scale research might become possible in the nearest future through collaboration with several clinical centers.

It was shown that the shift in the VWF size distribution towards smaller multimers may lead to an increase in the SIPAct level at sufficiently high AVF flow rates (Fig 6A). It may be supposed that the efficacy reduction of antiplatelet therapy in haemodialysis patients could be related to this fact [35,82]. These therapies are based on common drugs that do not sufficiently block shear-induced activation pathway in platelets [20,83,84]. Moreover, a shift in the VWF distribution to smaller multimers has been observed in haemodialysis patients [85]. We suppose that the use of drugs capable of effectively blocking SIPAct should reduce the level of thrombotic complications in haemodialysis patients [86,87].

The SIPAct level should initiate thrombus formation in cases when the level exceeds a certain individual value. It seems promising to conduct experiments aimed at the systematic investigation of the interpatient threshold variability in haemodialysis patients [20,88].

We suppose that the SIPAct level in the AVF is one of the risk factors for thromboembolic complications in haemodialysis patients [33]. Platelets move downstream from their priming location after passing the overcritical shear stress zone in a fistula. As a result, primed platelets are capable of provoking thromboembolic complications in distal vascular networks [16,19,20,89].

Acoustic diagnostic methods are of great interest to reduce the level of thrombotic complications in haemodialysis patients. To date, ultrasound methods have been applied not only for the acquisition of information on biomechanical AVF features (vessel geometry and flow waveforms) [44] but also for the direct detection of the initiation of thrombus formation [90]. Estimation of the SIPAct level in combination with the abovementioned methods may reduce the level of life-threatening complications in haemodialysis patients via timely antithrombotic intervention.

Arterial pressure rise leads to an increase in the AVF flow rate and consequently in the value of cumulative shear stress. In accordance with the current work, these changes should lead to an increase in the SIPAct level in a fistula. In contrast, a decrease in blood flow should reduce the cumulative shear stress. Moreover, a decrease in the blood flow rate below the threshold level should lead to the absence of SIPAct. From this point of view, even short-term physical activity or severe emotional stress could be additional risk factors for thrombotic complications in haemodialysis patients. The relationship of these factors with a thrombotic complication risk increase has been established for the general population [91,92]. It is of interest to investigate the role of SIPAct, which can be initiated by pressure spikes, in the occurrence of thrombotic complications in haemodialysis patients.

The current approach enables us to estimate the critical flow rate in patient-specific AVFs at the given VWF multimer size (Fig 7). Thus, an opportunity for the estimation of patient-specific safe blood pressure is created. The assessment of the indicated individual blood pressure level can be of clinical interest.

## Conclusion

In this paper, a patient-specific approach for the estimation of the SIPAct level in AVFs was developed. This approach is based on a combination of modern medical imaging technologies, CFD methods, and the mathematical model of platelet activation induced by unfolding of the VWF multimers [24,26]. The *in silico* approach may be applied for the determination of specific ways of thrombotic complication reduction in haemodialysis patients considering their individual features.

## Supporting information

S1 Text Estimation of the discretization error. (PDF)

S2 Text Parameter values and patient-specific data. (PDF)

S3 Text Flow rate waveforms. (PDF)

S4 Text Duration of computational experiments. (PDF)

S5 Text Limitations. (PDF)

S6 Text Additional results. (PDF)
