# Supplementary material for: Patient specific approach to analysis of shear-induced platelet activation in haemodialysis arteriovenous fistula
Source: PLoS One. 2022 Oct 3;17(10):e0272342. doi: 10.1371/journal.pone.0272342 (PMC9529124; doi:10.1371/journal.pone.0272342)
Supplement: S1 Text — (PDF) [file pone.0272342.s001.pdf]

S1 Text. Estimation of the discretization error

Estimation of the discretization error was conducted via the grid convergence index method [S1.1]. The quantity of interest was the platelet activation level (Equation (6) in the main text) in AVF P2 at the largest parameter values (Q_{in}^{a} = 1350 mL/min, *N* = 100). The results are listed in Table S1.1. The discretization error in the finest mesh solution was 6.4 %. This mesh was applied in the calculations. The computational mesh for AVF P1 contained approximately the same number of cells (931326).

Table S1-1. Discretization error of platelet activation level.

$M_1; M_2; M_3$	955343; 458700; 193424
$r_{21};\ r_{32}$	1.271; 1.333
$PAL_1\%;\ PAL_2\%;\ PAL_3\%$	0.191; 0.157; 0.146
p	6.24
PAL_{21}^{ext}	0.02
$e_a^{21}, \%$	17.8
$e_{ext}^{21}, \%$	4.9
$GCI_{fine}^{21}, \%$	6.4

References

S1.1. Celik IB, Ghia U, Roache PJ, Freitas CJ. Procedure for estimation and reporting of uncertainty due to discretization in CFD applications. J Fluids Eng. 2008;130(7):078001. doi: 10.1115/1.2960953.
