# Supplementary material for: Patient specific approach to analysis of shear-induced platelet activation in haemodialysis arteriovenous fistula
Source: PLoS One. 2022 Oct 3;17(10):e0272342. doi: 10.1371/journal.pone.0272342 (PMC9529124; doi:10.1371/journal.pone.0272342)
Supplement: S2 Text — (PDF) [file pone.0272342.s002.pdf]

S2 Text. Parameter values and patient-specific data

Table S2-1. Parameter values used in calculations.

Parameter	Range	Used value	Dimension	Comment number
η	$2.95 - 3.77$	3.16	cP	1
ρ	$1.042 - 1.06$	1.056	$\mathrm{g \cdot cm^{-3}}$	2
$\tau_{\#}(4)$	$32 - 170$	304	$\mathrm{dyn \cdot cm^{-2}}$	3
$\tau_{\#}(10)$		235		
$\tau_{\#}(20)$		175		
$\tau_{\#}(40)$		123		
$\tau_{\#}(60)$		99		
$\tau_{\#}(80)$		84		
$\tau_{\#}(100)$		74		
α	-	3.9	$\mathrm{dyn \cdot s \cdot cm^{-2}}$	4
k	-	-	1/s	5
$\mathrm{P_0}$	$(1.7 - 2.3) \cdot 10^5$	$2 \cdot 10^5$	$\mathrm{\mu L^{-1}}$	6
m	-	3	-	-

1. The range was calculated via the dependence of the dynamic viscosity on the haematocrit published elsewhere [S2.1]. The range of haematocrit for haemodialysis patients was retrieved from the literature [S2.2].

2. The range of density was retrieved from the literature [S2.3,S2.4].

3. The threshold shear stress $\tau_{\#}$ (Equation (1) in the main text) was calculated with the following expression:

$$\tau_{\#} = \eta \cdot \dot{\gamma}_0 \cdot \tilde{\tau}_{\#} \quad \text{(S2.1)}$$

where η denotes the dynamic viscosity of patient blood, and the value of $\dot{\gamma}_0$ was previously estimated as $6.75 \cdot 10^4$ 1/s (Supplementary information to [S2.5]). The dimensionless threshold shear stress $\tilde{\tau}_{\#} \equiv \tilde{\tau}_{\#}(N)$ for a given von Willebrand factor (VWF) multimer size N was calculated via a previously published procedure (S4 text to [S2.6]). According to *in vitro* experiments, SIPAct in platelet-rich plasma or whole blood is initiated at a shear rate varying between 1000 1/s and 5400 1/s [S2.7-S2.11]. The range of $\tau_{\#}$ values was obtained via multiplication of these values with the patient dynamic viscosity (η = 3.16 cP).

4. The threshold cumulative shear stress CSS_0 (Equation (1) in the main text) was calculated with the following equation:

$$CSS_0 = \alpha \cdot \widetilde{CSS}_0 \quad \text{(S2.2)}$$

where α is a dimensional factor and $\widetilde{CSS}_0$ denotes the dimensionless threshold of the cumulative shear stress. The dependence of $\widetilde{CSS}_0$ on the VWF multimer size was derived previously [S2.6]:

$$\widetilde{CSS}_0 = \left(\frac{3}{2}N\right)^{1/3} \sqrt{\frac{7}{4} + \frac{1}{4}\left(\frac{3}{2}N\right)^{-8/3} - 2\left(\frac{3}{2}N\right)^{-1/3}} \quad \text{(S2.3)}$$

According to *in vitro* experiments, the threshold of the cumulative shear stress ranges from 12 to 32 $\mathrm{dyn \cdot s/cm^2}$ (η = 3.16 cP) [S2.9,S2.11-S2.13]. The values of CSS_0 in this work approximately lie within this range at α equal to 3.9 $\mathrm{dyn \cdot s/cm^2}$.

5. Calculations were performed under the assumption that the value of *k* is sufficiently large to ensure the transition of all platelets to a priming state under overcritical cumulative shear stress.

6. The resting platelet concentration for haemodialysis patients was retrieved from the literature [S2.14,S2.15].

Table S2-2. Demographic and clinical data of patients at the time of magnetic resonance angiography.

**Parameter**	**Patient 1 (P1)**	**Patient 2 (P2)**
Age (years)	55	22
Gender	Female	Female
AVF age (years)	17	2
Cause of ESRD [1]	Polycystic kidney disease	Glomerulonephritis
RBC/HPF (count)	4	4
PLT ($10^3 \mu L^{-1}$)	158	255
Unfractionated heparin (during haemodialysis), IU	5000	7000
APTT (s)	28	28
Fibrinogen (g/L)	Within normal range	Within normal range
Prothrombin index (%)	Within normal range	Within normal range
Antiplatelet therapy	No	No
Haemorrhagic complications	No	No

[1] ESRD – end stage renal disease

References

S2.1. Guyton AC, Hall JE. Textbook of Medical Physiology. Eleventh edition. Philadelphia, USA: Elsevier Saunders; 2006.

S2.2. Caroli A, Manini S, Antiga L, Passera K, Ene-Iordache B, Rota S, et al. Validation of a patient-specific hemodynamic computational model for surgical planning of vascular

access in hemodialysis patients. Kidney Int. 2013;84(6):1237–45. doi: 10.1038/ki.2013.188.

S2.3. Kenner T. The measurement of blood density and its meaning. Basic Res Cardiol. 1989;84(2):111-24. doi: 10.1007/BF01907921.

S2.4. Carroll JE, Colley ES, Thomas SD, Varcoe RL, Simmons A, Barber TJ. Tracking geometric and hemodynamic alterations of an arteriovenous fistula through patient-specific modelling. Comput Methods Programs Biomed. 2020;186:105203. doi: 10.1016/j.cmpb.2019.105203.

S2.5. Zlobina KE, Guria GT. Platelet activation risk index as a prognostic thrombosis indicator. Sci Rep. 2016;6:30508. doi:10.1038/srep30508.

S2.6. Pushin DM, Salikhova TY, Zlobina KE, Guria GT. Platelet activation via dynamic conformational changes of von Willebrand factor under shear. PloS One. 2020;15:e0234501. doi: 10.1371/journal.pone.0234501.

S2.7. Ruggeri ZM. Mechanisms of shear-induced platelet adhesion and aggregation. Thromb Haemost. 1993;70(1):119–23. doi: 10.1055/s-0038-1646171.

S2.8. Miyazaki Y, Nomura S, Miyake T, Kagawa H, Kitada C, Taniguchi H, et al. High shear stress can initiate both platelet aggregation and shedding of procoagulant containing microparticles. Blood. 1996;88(9):3456–64. doi: 10.1182/blood.V88.9.3456.bloodjournal8893456.

S2.9. Holme PA, Ørvim U, Hamers MJ, Solum NO, Brosstad FR, Barstad RM et al. Shear-induced platelet activation and platelet microparticle formation at blood flow conditions as in arteries with a severe stenosis. Arterioscler Thromb Vasc Biol. 1997;17(4):646–53. doi: 10.1161/01.ATV.17.4.646.

S2.10. Lee H, Kim G, Lim C, Lee B, Shin S. A simple method for activating the platelets used in microfluidic platelet aggregation tests: Stirring-induced platelet activation. Biomicrofluidics. 2016;10(6):064118. doi: 10.1063/1.4972077.

S2.11. Rahman SM, Eichinger CD, Hlady V. Effects of upstream shear forces on priming of platelets for downstream adhesion and activation. Acta Biomater. 2018;73:228–35. doi:10.1016/j.actbio.2018.04.002.

S2.12. Ramstack JM, Zuckerman L, Mockros LF. Shear-induced activation of platelets. J Biomech. 1979;12(2):113–25. doi: 10.1016/0021-9290(79)90150-7.

S2.13. Bluestein D, Niu L, Schoephoerster RT, Dewanjee MK. Fluid mechanics of arterial stenosis: relationship to the development of mural thrombus. Ann Biomed Eng. 1997;25(2):344–56. doi: 10.1007/BF02648048.

S2.14. Hakim RM, Schafer AI. Hemodialysis-associated platelet activation and thrombocytopenia. Am J Med. 1985;78(4):575-80. doi: 10.1016/0002-9343(85)90398-5.
S2.15. Gafter U, Bessler H, Malachi T, Zevin D, Djaldetti M, Levi J. Platelet count and thrombopoietic activity in patients with chronic renal failure. Nephron. 1987;45(3):207-10. doi: 10.1159/000184118.
