# Supplementary material for: Patient specific approach to analysis of shear-induced platelet activation in haemodialysis arteriovenous fistula
Source: PLoS One. 2022 Oct 3;17(10):e0272342. doi: 10.1371/journal.pone.0272342 (PMC9529124; doi:10.1371/journal.pone.0272342)
Supplement: S3 Text — (PDF) [file pone.0272342.s003.pdf]

FS3 Text. Flow rate waveforms

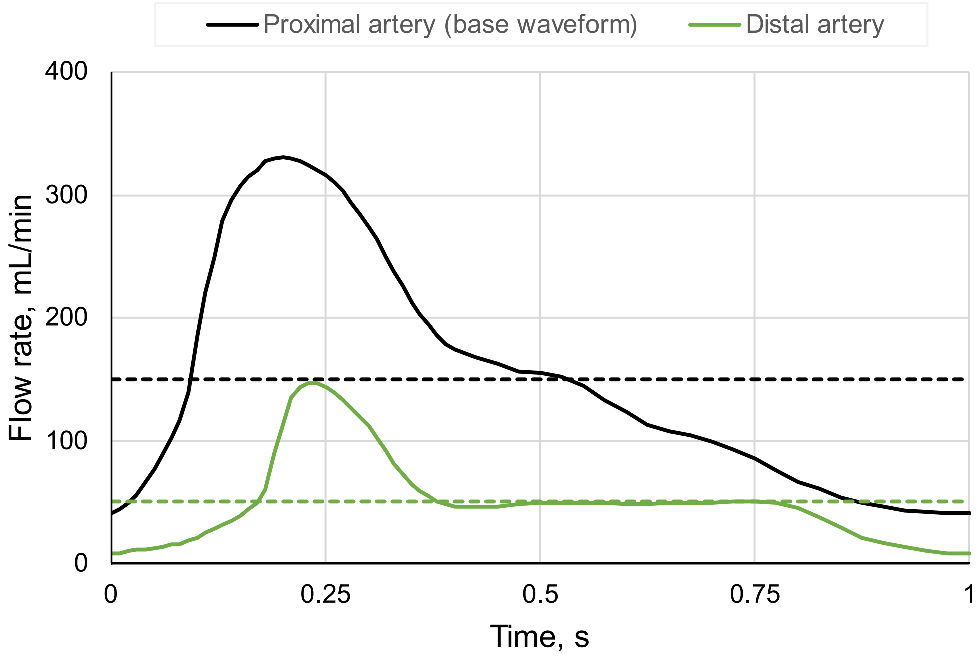

Fig S3-1. The time-dependent volumetric flow rate waveforms prescribed at the arterial inlet and outlet. The base flow rate waveform at the arterial inlet (Γ_{in}^{a}, Fig 2 in the main text) is shown (black curve). This waveform was adjusted by the translation along the OY axis to obtain the desired average flow rate per cardiac cycle (dashed black line) (Q_{in}^{a}). The flow rate waveform at the arterial outlet was similar in all calculations (green curve). The flow rate waveforms were adapted from the literature [S3.1].

References

S3.1. Ene-Iordache B, Remuzzi A. Disturbed flow in radial-cephalic arteriovenous fistulae for haemodialysis: low and oscillating shear stress locates the sites of stenosis. Nephrol Dial Transplant. 2012;27(1):358–68. doi: 10.1093/ndt/gfr342.
