# Supplementary material for: Patient specific approach to analysis of shear-induced platelet activation in haemodialysis arteriovenous fistula
Source: PLoS One. 2022 Oct 3;17(10):e0272342. doi: 10.1371/journal.pone.0272342 (PMC9529124; doi:10.1371/journal.pone.0272342)
Supplement: S4 Text — (PDF) [file pone.0272342.s004.pdf]

S4 Text. Duration of computational experiments

Table S4-1. Duration of computational experiments. Q_{in}^{a} means the average volumetric flow rate per cardiac cycle at arterial inlet (Γ_{in}^{a}, Fig 2 in the main text). The duration of one cardiac cycle was 1 s (Fig S3-1 in S3 Text). The data from averaging period was used at calculations of the SIPAct level (Equation (6) in the main text).

**AVF P1**			**AVF P2**		
Q_{in}^{a}, mL/min	Duration, s	Averaging period	Q_{in}^{a}, mL/min	Duration, s	Averaging period
775	6	3-6	1350	10	7-10
625	6	3-6	1200	10	7-10
488	10	7-10	1050	10	7-10
350	10	7-10	900	10	7-10
325	10	7-10	750	12	9-12
300	10	7-10	600	12	9-12
250	10	7-10			
200	10	7-10			
150	15	12-15			
