# Supplementary material for: Patient specific approach to analysis of shear-induced platelet activation in haemodialysis arteriovenous fistula
Source: PLoS One. 2022 Oct 3;17(10):e0272342. doi: 10.1371/journal.pone.0272342 (PMC9529124; doi:10.1371/journal.pone.0272342)
Supplement: S5 Text — (PDF) [file pone.0272342.s005.pdf]

## S5 Text. Limitations

The distensibility of vessel walls was not considered in the calculations of the SIPAct level. It is likely that the SIPAct level in the AVF is overestimated in rigid-wall simulations. This work demonstrates that decreasing the flow rate and, as a consequence, the shear stress leads to a decrease in the SIPAct level in the AVFs. In turn, studies have indicated that accounting for AVF vessel motion leads to a decrease in the wall shear stress by 20–50% below the results of rigid-wall calculations [S5.1,S5.2]. Thus, considering distensibility should lead to decreasing shear stress and SIPAct levels in AVFs. In this regard, the current work describes the approach enabling to calculate the upper bound of the SIPAct level *in vivo*. In particular, a sufficient no-activation condition may be obtained (Fig 7 in the main text).

The influence of the flow rate on the SIPAct level in the AVF was investigated under the assumption of a constant flow rate through the arterial outlet ($\Gamma_{out}^{a}$, Fig 2 in the main text). The AVF forms a low-resistance circuit in parallel with a high-resistance circuit, including small vessels of the lower arm [S5.3]. Following electrical analogies of the mechanical system, an increase in the flow rate through the arterial inlet should slightly affect the flow rate through the arterial outlet.

The VWF size distribution was assumed to be monodisperse instead of polydisperse, normally detected *in vivo*. In this regard, certain notions should be made regarding the application of this approach in practical settings. Only multimer sizes above the threshold value should be used in calculations [S5.4]. The existence of a critical VWF multimer size has been suggested by several authors [S5.5-S5.9]. Within this context, a reasonable way of using the current approach is to estimate the SIPAct level at critical and maximal VWF multimer sizes detected in patient plasma. It may be supposed that the SIPAct level in the AVF should occur within the abovementioned range, as the size of VWF multimers correlates with the SIPAct level *in vitro* [S5.10-S5.12]. Contradictions in data on the critical VWF size occur [S5.6,S5.7,S5.9]. This problem requires further investigation.

### References

S5.1. Decorato I, Kharboutly Z, Vassallo T, Penrose J, Legallais C, Salsac AV. Numerical simulation of the fluid structure interactions in a compliant patient-specific arteriovenous fistula. Int J Numer Method Biomed Eng. 2014;30(2):143–59. doi: 10.1002/cnm.2595.

S5.2. McGah PM, Leotta DF, Beach KW, Aliseda A. Effects of wall distensibility in hemodynamic simulations of an arteriovenous fistula. Biomech Model Mechanobiol. 2014;13(3):679–95. doi: 10.1007/s10237-013-0527-7.

S5.3. Dixon BS. Why don't fistulas mature? Kidney Int. 2006;70(8):1413–22. doi: 10.1038/sj.ki.5001747.

S5.4. Zlobina KE, Guria GT. Platelet activation risk index as a prognostic thrombosis indicator. Sci Rep. 2016;6:30508. doi: 10.1038/srep30508.

S5.5. Slepian MJ, Sheriff J, Hutchinson M, Tran P, Bajaj N, Garcia JG et al. Shear-mediated platelet activation in the free flow: Perspectives on the emerging spectrum of cell mechanobiological mechanisms mediating cardiovascular implant thrombosis. J Biomech. 2017;50:20–5. doi: 10.1016/j.jbiomech.2016.11.016.

S5.6. Andrews RK, López J, Berndt MC. Molecular mechanisms of platelet adhesion and activation. Int J Biochem Cell Biol. 1997;29(1):91–105. doi: 10.1016/S1357-2725(96)00122-7.

S5.7. Stockschlaeder M, Schneppenheim R, Budde U. Update on von Willebrand factor multimers: focus on high-molecular-weight multimers and their role in hemostasis. Blood Coagul Fibrinolysis. 2014;25(3):206–16. doi: 10.1097/MBC.0000000000000065.

S5.8. Mody NA, King MR. Platelet adhesive dynamics. Part II: high shear-induced transient aggregation via GPIbα-vWF-GPIbα bridging. Biophys J. 2008;95(5):2556–74. doi: 10.1529/biophysj.107.128520.

S5.9. Parker ET, Lollar P. Conformation of the von Willebrand factor/factor VIII complex in quasi-static flow. J Biol Chem. 2021;296:100420. doi: 10.1016/j.jbc.2021.100420.

S5.10. Moake JL, Turner NA, Stathopoulos NA, Nolasco LH, Hellums JD. Involvement of large plasma von Willebrand factor (vWF) multimers and unusually large vWF forms derived from endothelial cells in shear stress-induced platelet aggregation. J Clin Investig. 1986;78(6):1456–61. doi: 10.1172/JCI112736.

S5.11. Zlobina KE, Guria GT. Platelet activation risk index as a prognostic thrombosis indicator. Sci Rep. 2016;6:30508. doi: 10.1038/srep30508.

S5.12. Zhang C, Kelkar A, Neelamegham S. von Willebrand factor self-association is regulated by the shear-dependent unfolding of the A2 domain. Blood Adv. 2019;3(7):957–68. doi: 10.1182/bloodadvances.2018030122.
