# Supplementary material for: Patient specific approach to analysis of shear-induced platelet activation in haemodialysis arteriovenous fistula
Source: PLoS One. 2022 Oct 3;17(10):e0272342. doi: 10.1371/journal.pone.0272342 (PMC9529124; doi:10.1371/journal.pone.0272342)
Supplement: S6 Text — (PDF) [file pone.0272342.s006.pdf]

S6 Text. Additional results

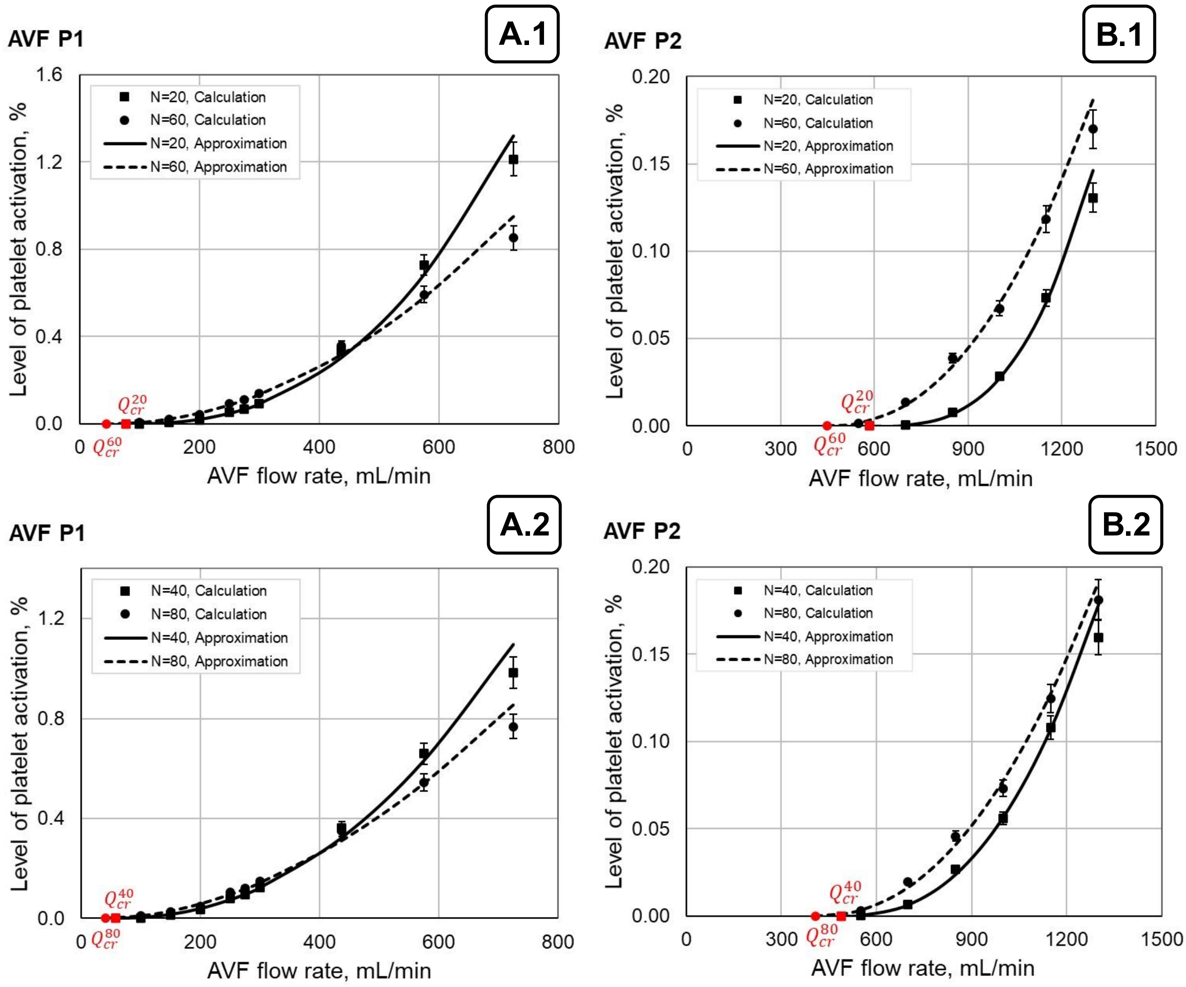

Fig S6-1. The dependence of the SIPAct level (Equation (6) in the main text) on AVF flow rate (Equation (9) in the main text) in P1 (column A) and P2 (column B) AVFs. The results in both fistulas were obtained at VWF multimers sizes equal to 20, 60 (A.1, B.1) and 40, 80 (A.2, B.2). The results were approximated by Equation (10) in the main text.

Table S6-1. The founded parameter values of the approximation curves (Equation (10) in the main text).

	AVF P1			AVF P2		
N	a, %	β	Q_{cr}, mL/min	a, %	β	Q_{cr}, mL/min
4	–	–	143.0	–	–	925
10	$2.029E-08$	2.809	99.7	$5.253E-10$	2.989	736
20	$1.214E-07$	2.503	78.0	$2.277E-10$	3.085	584
40	$8.307E-07$	2.167	58.0	$1.070E-08$	2.483	489
60	$2.130E-06$	1.994	45.0	$4.449E-08$	2.260	448
80	$4.747E-06$	1.853	40.0	$6.506E-08$	2.192	407
100	$6.990E-06$	1.778	33.0	$6.166E-07$	1.849	397

The dependence of the threshold flow rate Q_{cr} on VWF multimers size N (Fig 7 in the main text) was approximated via the following equation:

$$Q_{cr} = \delta \cdot N^{\gamma} \tag{S6.1}$$

The founded values of parameters were $\delta_1 = 277.883$ mL/min, $\gamma_1 = -0.444$ for AVF P1 and $\delta_2 = 1341.173$ mL/min, $\gamma_2 = -0.269$ for AVF P2.

Supporting movies

https://guria-lab.ru/media/PLOSONE-2021/AVFP1_tau.mp4

https://guria-lab.ru/media/PLOSONE-2021/AVFP2_tau.mp4

https://guria-lab.ru/media/PLOSONE-2021/AVFP1_CSS.mp4

https://guria-lab.ru/media/PLOSONE-2021/AVFP2_CSS.mp4

https://guria-lab.ru/media/PLOSONE-2021/AVFP1_Pa.mp4

https://guria-lab.ru/media/PLOSONE-2021/AVFP2_Pa.mp4
